# Supplementary material for: Molecular phylogeny and evolutionary history of Moricandia DC (Brassicaceae)
Source: PeerJ. 2017 Oct 27;5:e3964. doi: 10.7717/peerj.3964 (PMC5661452; doi:10.7717/peerj.3964)

Molecular phylogeny and evolutionary history of *Moricandia* DC (Brassicaceae)

Francisco Perfectti, José M. Gómez, Adela González-Megías, Mohamed Abdelaziz and Juan Lorite

Supplementary information

Table S1: GenBank accessions

Table S2: Comparison between the proposed new species *Moricandia rytidocarpoides* and *Rytidocarpus moricandioides* (N. Africa) for several quantitative morphological traits.

Table S3: Comparison of the proposed new species *Moricandia rytidocarpoides* with the most related species within the genus and with *Rytidocarpus moricandioides* from North Africa.

Table S4: Additional examined specimens.

Figure S1: Maximum likelihood tree produced with the complete 24-samples set. Branch labels represent bootstrap percentage values. Scale: mean expected rates of substitution per site.

Figure S2: Tanglegram showing the maximum likelihood inference trees obtained from cpDNA (ndhF + trnT-trnF) and nuclear (ITS1 + ITS2) sequences.

Figure S3: Biplots showing the result of non-metric multidimensional scaling (NMDS) for individuals (left) and variables (right) performed with the morphological data presented in Supplementary table 2. Model data in the left are referred to the result of a Permutational Multivariate analysis using species as factor.

Figure S4: Microphotography of fruits of *M. rytidocarpoides* (left) *and R. moricandioides* (right). Fruits (a and b) and detail of fruit valve (c and d). Central insert (c) shows size differences of the fruit.

Table S1. GenBank accessions

Taxon	Sample	GenBank accessions		
		ITS	*ndhF*	*trn*T-*trn*F
Eruca pinnatifida	Erupinn01-15-01	MF192766	MF192790	MF192814
	Erupinn02-15-01	MF192767	MF192791	MF192815
Eruca vesicaria	Eruves01-15-01	MF192768	MF192792	MF192816
Moricandia arvensis	Mar01-14-01	MF192771	MF192795	MF192819
	Mar33-05-01	MF192772	MF192796	MF192820
	Mar35-13-01	MF192773	MF192797	MF192821
	Mar38-05-01	MF192774	MF192798	MF192822
	Mar42-05-01	MF192775	MF192799	MF192823
Moricandia suffruticosa	Msu01-14-01	MF192776	MF192800	MF192824
Moricandia foetida	Mfo01-14-01	MF192788	MF192812	MF192836
	Mfo02-13-01	MF192789	MF192813	MF192837
Moricandia foleyi	Mfy01-15-01	MF192769	MF192793	MF192817
	Mfy02-15-01	MF192770	MF192794	MF192818
Moricandia moricandioides baetica	Mmob01-14-01	MF192779	MF192803	MF192827
Moricandia moricandioides gienensis	Mmog06-12-01	MF192780	MF192804	MF192828
Moricandia moricandioides moricandioides	Mmom05-12-01	MF192781	MF192805	MF192829
Moricandia moricandioides pseudofoetida	Mmsf01-15-01	MF192782	MF192806	MF192830
	Mmsf02-15-01	MF192783	MF192807	MF192831
Moricandia spinosa	Mspi01-14-01	MF192777	MF192801	MF192825
Moricandia nitens	Mni03-14-01	MF192778	MF192802	MF192826
Rytidocarpus moricandiodes	Rmorm01-14-01	MF192787	MF192811	MF192835
	Rmorg02-14-01	MF192786	MF192810	MF192834
Moricandia rytidocarpoides	Rmorg01-13-01	MF192784	MF192808	MF192832
	Rmorg01-14-01	MF192785	MF192809	MF192833

Table S2. Comparison between the proposed new species *Moricandia rytidocarpoides* and *Rytidocarpus moricandioides* (from Morocco) for several quantitative morphological traits (mean±SE). P-values have been obtained after GLMs (using family Gaussian for continuous data and Poisson for counting). n.s. = not significant (P>0.05).

	M. rytidocarpoides		***R. moricandioides***		***P-value***
No. stems	1.13±0.06	(n=63)	2.29±0.57	(n=7)	**0.0178**
Plant height (cm)	31.12±1.76	(n=63)	43.78±4.33	(n=7)	**0.0251**
Lower leaves length (mm)	53.92±3.42	(n=63)	79.93±15.97	(n=7)	**0.0266**
Lower leaves width (mm)	14.58±1.44	(n=63)	38.02±5.37	(n=7)	**<0.0000**
Upper laves length (mm)	12.69±1.76	(n=63)	15.76±1.66	(n=7)	n.s.
Upper laves width (mm)	2.75±0.31	(n=63)	5.16±0.68	(n=7)	**0.0152**
No. flowers per inflorescence	7.9±0.3	(n=63)	11.5±1.45	(n=7)	**0.0034**
No. flowers per plant	60.17±5.97	(n=63)	128.14±44.5	(n=7)	**<0.0000**
Flower pedicel length (mm)	4.12±0.11	(n=63)	6.38±0.27	(n=7)	**<0.0000**
Sepal length (mm)	7.63±0.35	(n=51)	11.42±0.52	(n=7)	**<0.0000**
Sepal width (mm)	2.16±0.11	(n=51)	2.65±0.28	(n=7)	n.s.
Petal length (mm)	13.26±0.51	(n=51)	23.01±1.84	(n=7)	**<0.0000**
Petal width (mm)	3.87±0.21	(n=51)	8.18±1.53	(n=7)	**<0.0000**
Long stamen length (mm)	10.4±0.25	(n=51)	13.9±0.86	(n=7)	**<0.0000**
Short stamen length (mm)	8.40±0.24	(n=51)	11.08±0.95	(n=7)	**<0.0000**
Fruit length (mm)	13.64±0.18	(n=58)	24.45±0.82	(n=7)	**<0.0000**
Fruit width (mm)	3.20±0.11	(n=58)	4.25±0.50	(n=7)	**0.0075**
No. seeds per fruit	19.00±0.74	(n=37)	39.67±4.59	(n=6)	**<0.0000**
Seeds length (mm)	1.27±0.02	(n=39)	1.30±0.04	(n=6)	n.s.
Seed width (mm)	0.75±0.02	(n=39)	0.88±0.06	(n=6)	**0.0280**

Table S3. Comparison of the proposed new species *Moricandia rytidocarpoides* with the most related species within the genus and with *Rytidocarpus moricandioides* from North Africa.

	M. moricandioides moricandioides	***M. foetida***	***M. rytidocarpoides* (SE Spain)**	***R. moricandioides* (Africa)**
**Habit**	Annual to biannual (suffruticose at base)	Annual	Annual	Annual
**Plant height (cm)**	Up to 80	Up to 50	Up to 75	Up to 60
**Lower leaves**	Rosette, sub-sessile, obovate with sinuate-dentate margin<br>20-99 x 9-80 mm	Rosette forming, sub-sessile, obovate with dentate margin<br>30-75 x 20-40	Rosette forming, sub-sessile with lobate to pinnatifid margin<br>13-175 x 2-81	Rosette, ovate-oblongue, obtuse with sinuate to dentate margin<br>10-147 x 18-55
**Upper leaves (mm)**	Chordate-amplexicaule, acute to obtuse at the appex<br>10-40 x 10-30	Chordate-amplexicaule, markedly acute at the appex<br>12-24 x 8-15	Chordate to sagittate, amplexicaule, markedly acute at the appex<br>10-14 x 0.9-12	Chordate-amplexicaule, acute at the appex<br>13-18 x 4-8
**Inflorescence shape**	Racemose-no flexuose	Recemose-flexuose	Racemose, no flexuose	Racemose, no flexuose
**Nº flowers**	c. 60	c. 15	c. 60	c. 128
**Sepal**	Purple, corniculate at the appex, gibose at the base	Purplish, obtuse at the appex, gibose at the base	Green-light purplish, acute at the appex, gibose at the base	Purplish, acute at the appex, gibose at the base
**Sepal (mm)**	11.5-15 x2.2-4	8-10.2 x 1.5-2.4	7.1-10.3 x 1-2.5	9.3-13.1 x 2.3-3.9
**Petal (mm)**	c. 25(18-29)	c. 12 (9-20)	c. 13(7-23)	c. 23(14-28)
**Petal color**	Purple-purplish	White-light purplish	Light purplish	Purple
**Fruit (mm)**	60-140 x 1.1-3	40-65 x 1.1-2.4	9.5-16 x 3-3.4	17-28 x 3.7-4.8
**Fruit pedicel (mm)**	c. 6.5	c. 10	c. 4.1	c. 6.4
**Fruit valve**	Smooth with 1 nerve	Smooth with 1 nerve	Markedly reticulate with 1 nerve	3-5 nerves
**Seeds**	Uniseriate at each locule<br>Narrowly alate	Uniseriate<br>Narrowly alate	Biseriate<br>Narrowly alate	Biseriate<br>Narrowly alate
**Seeds per fruit**	32 (21-52)	29 (19-40)	19(11-30)	40(28-58)
**Seed size (mm)**	1.2-2.4 x 1-1.4	1.3-1.8 x 10.-1.3	1.1-1.65 x 0.4-1.1	1.2-1.5 x 0.8-1.1
**Habitat**	Dry and semiarid areas over marls, clays and limestones	Semiarid hills over marls often with gypsum	Dry and semiarid hills over marls	Dry crop fields and hills over marls
**Alt. range (m)**	300-700	0-400	400-800	250-700

Table S4. Additional examined specimens.

Eruca foleyi stat. nov. (Sub. Moricandia foleyi **Batt.):** MOROCCO: c. Ain Zora, Mtalsa, GDA28422, 850 m, 1929-10-26, Font Quer; prox. Merzouga, 700 m, 31º3.5' N / 4º 0.7 W, ramblas arenosas desérticas, GDA62593**,** 12/04/2014, M. Abdelaziz, M.J. Gómez, J. Lorite & F. Perfectti**;** Prox. Merzouga, 700 m, 31º3.5' N / 4º 0.7 W, Ramblas arenosas desérticas, GDA62594, 18/02/ 2014, M. Abdelaziz, M.J. Gómez, J. Lorite & F. Perfectti **;** Carretera entre Rissani y Merzouga, 700 m, 31º16.9' N / 4º 16.5' W, Ramblas arenosas desérticas, GDA62595**,** 12/04/ 2014, M. Abdelaziz, M.J. Gómez, J. Lorite & F. Perfectti.

Moricandia arvensis **(L.) DC.:** FRANCE: Éze s/mer (AM), 1964-01-06, MA, MA194666, Gavelle; ISRAEL: Southern Negev, 8 Km SE of Mizpe Ramon, Nahal Gevanim, 1989-03-29, SEV218603, F. Amich, A. Danin, S.G. Gardner & B. Valdés; ITALY: Nebrodi: Portella Femmina Morta, 37.9º N / 14.48 º W, 1550 m, 1990-06-09, SEV254568, F.M. Raimondo, S.L. Jury, R.M. Gebauer, A. Charpin, S. Brullo et al. MOROCCO: Ouezzane. Mjara, entre Ain Dorij y Teroual, 34.64 º N / -5.28º W, 160 m, 2004-05-22, SEV251241, Márquez, F.J. Pina, C. Santa-Bárbara & B. Valdés; Er Rachidia road N of Erfoud, 59 kms from Rissani, 1 km N of Ksar Jdid, 31.73º N / -4.19º W, 915 m, 2002-02-19, SEV240385, Jury et al. PORTUGAL: Silves, laderas castillo, 2011-02-26, SEV270303, E. Sánchez Gullón; SPAIN: Alicante: La Nucía, bajada a la Acequia Máre, GDA17058, 1984-11-18, L. Cano; Alicante: Santapola. GDA45717, L. Garcia-Vicente; Almería: Lúcar, Pocicos, GDAC43378, 1995-6-3. Fco. Navarro Reyes; Almería: Lúcar, Rambla Lúcar, GDAC43388, 1995-7-23, Fco. Navarro Reyes; Almería: Lúcar, Montroy, GDAC43419, 1996-5-11, Fco. Navarro Reyes; Almería: Playa de Carboneras, GDAC40287, 1996-3-23, Eduardo Linares; Almería: Rambla de Paulenca, GDAC15771, 1983-1-30, M. Cueto; Almería: Rambla antes de Retamar, GDAC 15712, 1983-1-2, M. Cueto; Almería: Níjar, La Joya, GDA26054, 150 m, 1990-12-16, M.J.M. Lirola & L. Gutiérrez; Almería: Alhama de Almería: GDAC29177, 450 m, 1988-11-14, G. Blanca; Almería: Enix, barranco del Palmar, GDA45718, 80 m, J. M. Román Díaz; Almería: Sª Almagrera, GDA50110, 162 m, 2005-4-14, C. Morales, C. Quesada, L. Baena & M.T. Vizoso; Almería: Carboneras. GDA26052, 5 m, 1991-6-1, M.J.M. Lirola & L. Gutiérrez; Almería: pasado Sorbas, Los Castaños, GDA51610, 356 m, 2006-3-28, C. Morales, M.T. Vizoso & G. Muñoz; Barcelona: Vallès occidental, Castellbisbal, cruce de carreteras Castellbisbal-Rubí-Molins, GDA48389, 2002-4-8, J. Vicens; Granada: Alamedilla, río Guadahortuna, GDAC40565, 800 m, 1995-7-2, C. Salazar; Granada: Baza, Barranco del Espartal, 800, 37º31.2' N / 2º 42.2’ W, GDA 62592, Taludes margosos, 01/07/2016, A. Caravantes; Granada: Pedro Martínez, Mencal, Morrón Artero, GDA25107, 1050 m, 1991-6-26, R. Sánchez-Pérez; Granada: carretera Illora-Montefrío, GDA16996, 850 m, 1982-6-25, Aroza, Socorro, Negrillo; Granada: Rambla de Albuñol, cruce de la Rábita, GDAC19736, 1982-2-18, A.B. Robles & al.; Granada: entre Guadix y Purullena, GDAC19738, 1984-4-11, A.B. Robles & al.; Granada: Río Darro, debajo del Cerro del Sol, GDAC23282, 1984-7-13, A.B. Robles & al.; Granada: Guadix, Baños de Alicún, GDA50222, 850 m, 2003-6-15, M.N. Jiménez; Jaén: Sª de Alta Coloma, bajada del Puerto de las Palomas, GDAC35419, 1000 m, 1990-2-28, Pablo Navarro; Jaén: Fundición "La Cruz" de Linares, GDAC20916, 1982-4-6, J.C. Trujillo; Jaén: entre Jaén y Pegalajar, pr. Granja del huevo, GDA45723, 500 m, G. Blanca, C. Morales & C. Díaz de la Guardia; Madrid: Alcalá de Henares, 690 m, 1974-04-20, SEV41053, A. Segura-Zubizarreta; Málaga: entre Coín y Monda, GDA13276, 1977-3-20, F. Pérez Raya; Murcia: Jumilla. Finca La Esperanza, 1992-04-18, SEV218601, Z. Díaz; Murcia: Puerto de Lumbreras, Rambla del Nogalete, GDA6157, 1970-6-24, L. Carreras & E. Valdés; Palma de Mallorca: Porto Pi, GDA, 28424, 1947-7-29, Palau Ferrer; Toledo: La Guardia, cerros del arroyo Cedrón, GDA16356, 680 m, 1982-10-17, S. Laorga; Valladolid: Parquesol, 41.63ºN / -4.76 º W, 1999-09-01, MA694116, J.P. Del Monte. TUNISIA: Djebel-Djeloud, 1907-01-01, 1907 m, MA50266, C.J. Pitard.

Moricandia foetida **Bougeau *ex* Cosson.** SPAIN: Almería: Alsodux, GDAC41675, 1996-5-10, L. Baena; Almería: Tabernas, Llanos de Rueda, GDAC40288, 340 m 1996-3-22, Eduardo Linares; Almería: Campos de Tabernas, GDAC35766, 1990-3-9, C. Morales & al.; Almería: Tabernas, desierto de Tabernas, rambla del Búho, GDA49837, 335 m, 2004-4-6, J. Peñas; Almería: Tabernas, GDA47546, 340 m, 2003-3-29, H. Blanca; Almería: Rioja-Tabernas, GDA43587, G. Blanca, M. Cueto & M.J. Martínez; Almería: prox. de Alsodux, GDAC42352, 380 m, 1996-4-27, J. Lorite; Almería: Campos de Tabernas, GDAC14376, 1983-2-27, C. Morales; Almería: Mini-Hollywood, GDAC15711, 1983-4-26, J. Guirado; Almería: Tabernas, cerca de la Central solar, GDAC26709, 1987-3-18, C. Morales & A.T. Romero; Almería: de Tabernas a Gérgal, GDA6158, 1972-5-1, E. Valdés-Bermejo; Almería: cuevas de Almanzora, Los silos de Vera, GDA45713, 80 m, M. J. Salinas & J. Peñas; Almería: Sª Alhamilla, en la base, Campos de Tabernas, cerca de Mini Hollywood, GDAC31344, 1989-3-28, C. Morales & A.B. Robles-Cruz; Cartagena: Tallante, 1972-10-06, MA315049-2, Rivas, Esterel, Gómez & Valdés.

***Moricandia moricandioides* (Boiss.) Heywood subsp. *moricandioies*:** SPAIN: Almería: Sª de Gádor, Instinción, GDAC37320-2, 550 m, 1992-5-2, A. Hervás; Almería: Adra, Río Grande, GDAC28410, 1984-2-24, J. Guirado; Almería: entre Laujar y Paterna, GDA45761, 1989-6-14, C. Morales; Córdoba: Priego, GDA6159, 1972-4-30, E. Valdés Bermejo; Almería: prox. de Ohanes, GDA52073, 700 m, 1996-11-27, J. Lorite; Almería: prox. de Canjáyar, GDA52074, 690 m, 1996-11-27, J. Lorite; Granada: Órgiva, Sª de Lújar, proximidades a Barranco de los Castillejos, GDA19763, 400 m, 1987-4-17, O. Socorro & M.C. Espinar; Granada: carretera de Jaén a un km del cruce de Deifontes, GDA14627, 1982-4-14, J. Hurtado; Granada: Sª Sagra, Huéscar, Cortijos de la Umbría, GDA11568, 1460 m, 1977-10-16, A.M. Negrillo; Granada: Sª Lújar, cerca de Rules, GDAC19925, 1983-2-5, A.B. Robles & al.; Granada: Sacromonte, GDAC19924, 1984-7-13, A.B. Robles & al.; Granada: Alpujarra, Jorairata-Murtas, GDAC19927, 1984-3-22, J. Guirado; Granada: Galera, GDAC19928, 1984-8-9, A.B. Robles & al.; Granada: Alpujarra, entre Picena y Cherín, GDAC19929, 1984-8-4, A.B. Robles & al.; Granada: Alamedilla, GDA48298, 850 m, 2003-5-16, C. Morales, L. Baena & F.B. Navarro; Granada: alrededores de Colomera, antes de llegar al pueblo, GDA51575, 1999-3-30, C. Morales & col.; Granada: Dehesa del Generalife, Valle del río Darro, Vereda que va a Jesús del Valle, GDA51543, 914 m, 2001-3-31, C. Morales, L. Baena & R. Montiel; Granada: Loja, base del Hacho, GDA51540, 566 m, 2001-5-2, C. Morales, L. Baena & F.B. Navarro; Granada: Almegíjar, Bco. de las Tobas, de Cádiar a Torvizcón, km 41, primer barranco, orientación N, GDA51538, 742 m, 2001-4-5, C. Morales, C. Quesada, L. Baena & M.T. Vizoso; Granada: Almegijar, próx. al puente sobre el Guadalfeo, GDA9515, 600 m, 1976-2-27, J. Molero-Mesa; Granada: Baza, Barranco del Espartal, 780 m, 37º31.2' N / 2º 42.2 W, Taludes margosos, GDA6259, 01/07/2016, A. Caravantes; Jaén, cercanías de Solera, GDAC3752, 1977-5-31, J.A. Gil, G. Blanca, F. Valle y A. Ortega; Jaén, Puente Tablas, orillas del río Guadalbullón, GDA25591, 380 m, 1986-5-12, C. Fernández; Jaén: La Cerradura, GDAC27577, 700 m, 1988-4-16, G. Blanca, Madrid: carretera de Torres de la Alameda a Villalbilla, GDA16399, 1980-4-17, Barreno, Merino, Rico & Silva; Málaga: Torcal de Antequera, GDAC3970, 1977-4-15, J. Guerra; Sevilla: Morón de la Frontera, GDAC15910,1982-3-24 Ladero, F. Navarro, Pérez Chiscano & C. Valle.

***Moricandia moricandioides* subsp. *baetica* (Boiss. & Reut.) Sobrino-Vesperinas:** SPAIN: Almería, Sª de Gádor, El Atajo, 640 m, HUAL13863, 2005-04-20, F.J. Pérez-García, J.A. Garrido & J.M. Medina-Cazorla; Granada: Saladares de El Margen, GDAC42376, 1997-5-9, M.E. González-Giménez; Jaén: entre Úbeda y Baeza, GDA45763, 1988-3-29, A.T. Romero; Jaén: entre Úbeda y Baeza, 1988, GDA45763, A.T. Romero; Jaén: Pozo Alcón, Entre P. Alcon y Baza, 1991-06-11, MGC32614, J. M. Nieto & A. V. Pérez Latorre; Málaga: Cuevas de San Marcos, Arroyo Los Puercos, 561 m, 2008-05-17, MGC68234, B. Cabezudo, A. V. Pérez Latorre, O. Gavira & M. Becerra; Málaga: La Araña. Fábrica de cemento. Zona Este 7, 50 m, 2003-03-05, O. Gavira; Málaga: Antequera, Nacimiento de la Villa, MGC52098, B. Cabezudo; Málaga: Jimera de Líbar, Carretera hacia Atajate, 540 m, MGC51567, M. Becerra; Málaga: Archidona, Hoz de Marín, 2001-02-09, MGC47891, B. Cabezudo, A. V. Pérez Latorre & O. Gavira; Málaga: Ronda, Sierra de los Merinos, Carril que sale de la carretera Ronda - El Burgo en Venta Añoreta, 1996-05-10, MGC44677, B. Cabezudo & D. Navas; Málaga: Rincón de la Victoria, El Cantal, La Cala del Moral, 1974-02-23, MGC MGC1195, A. Asensi & B. D. Garretas;

***Moricandia moricandioides* subsp. *cavanillesiana* (Font Quer & A. Bolòs) Greuter & Burdet.** SPAIN: Lérida: Torcal de Montmareu. GDA7638, 1969-7-1, L. Carreras & E. Valdés Bermejo; Zaragoza: Caspe, laderas margosas sobre el Guadalupe, junto al puente de la carretera a Maella, 41.2ºN / 0.01 ºW, 140 m, 4-10-93, MA523438, V.J. Arán & Mª.J. Tohá; Zaragoza: Cerca de Osera, 1973-04-29, MA395272, J. Fernández-Casas.

***Moricandia moricandioides* subsp. *giennensis* Valdes**. SPAIN: Jaén: Colegio Universitario, GDA24437, 440 m, 1984-4-2, C. Fernández; Jaén: alrededores, GDA6168, 1972-4-29, E. Valdés Bermejo; Granada: entre la Malá y el Cruce de Escúzar, GDAC19923, 1984-4-9, A.B. Robles & al.; Granada, carretera Granada-Pantano de los Bermejales, GDAC19922, 1984-4-17, A.B. Robles; Granada, prox. a la Malahá, GDAC19921, 1982-4-8, A.B. Robles; Granada, Zújar, Guadiana Menor, GDAC19920, 1984-8-8, A.B. Robles & al.

***Moricandia moricandioides* subsp. *pseudofoetida* Sánchez-Gómez, M.A. Carrión, A. Hern. & J. Guerra**
SPAIN: Murcia, Embalse de los Rodeos, 38.07 ºN / 1.0 º W, 2009-03-16, MUB108945, P. Sánchez-Gómez, J. B. Vera & J.L. Cánovas; Murcia, Cehegin, La Atalaya, 37.973 ºN / 1.0 º W, 2000-06-05, MUB105866, P. Sánchez-Gómez; Murcia: Puerto del Garruchal, 37.954 ºN / 1.0 º W, 2003-03-25, MUB105856, M. A. Carrión, J. A. López & J. Moya; Murcia: Barranco de la Mina, 37.945 ºN / 1.20 º W, MUB105853, P. Sánchez

Gómez & M.A. Carrión; Murcia: Sangonera la Seca, Rambla de los Serranos, 37.945 º N / 1.0 º W, 2003-04-02, MUB105774, E. Rodriguez.

***Moricandia nitens* (Viv.) E.A. Durand & Barratte.** ISRAEL: Southern Negev, 6 Km S of Zihor junction, 390 m, 1989-03-29, SEV218604, F. Amich, A. Danin, S.G. Gardner & B. Valdés; Negev, Wadi Hurra, 1952-04-03, SEV51605, M. Zohary & J. D´Angelis. MOROCCO: Agouin, 1774 m., 31º10.12 'N 7º29.262' W. Matorral del *Hammada hispanica*. GDA62597**,** 10/04/2014. M. Abdelaziz, M.J. Gómez, J. Lorite & F. Perfectti. Det.: J. Lorite. TUNISIA: Gabès, Djebel Tebaga, base de las colinas a unos 8 Km al S de la carretera GP 16 Gabès Kebili, 33.78 º N / 9.63 º W, 150 m, 1992-05-24, SEV218095, C. Benedi, C. Blanché & J. Vallès.

***Moricandia rytidocarpoides* Lorite, Perfectti, Gómez, González-Megías & Abdelaziz *sp. nov*.** SPAIN: Jaén, Mancha Real, km 32 Jaén-Úbeda, 540 m., JAEN77586, 30SVGVG48, Carlos Fernández, 10-VI-1977; Ibidem JAEN77586; Jaén, Arroyo Salado, Margas y sales, 440 m., 30SVG4391, JAEN941812, C. Tornero, E. Piñero, E. Moreno et al., 21-V-1994; Jaén: Quesada, Lacra. Margas con yesos, 800 m, 30SVG9285, JAEN942162, L. Morillas, 10-IV-1994; Ibidem, JAEN942172; Ibidem, JAEN942173; Ibidem, JAEN942175; Ibidem, JAEN942187; Jaén: Quesada, Rambla de la Mina, Margas y sal, 480 m., 30SVG8884, JAEN942303, L. Morillas, 08-V-1994; Ibidem, JAEN942304; : Jaén, Quesada, Lacra, Margas con yesos, 800 m, 30SVG9285, L. Morillas, JAEN952074, 16-IV-1995; Ibidem JAEN952074.

***Moricandia spinosa* (Desf.) Coss.** MOROCCO: carretera Missour- Boulemane, 938 m., 33º2.144' N 4º4.083' W. GDA62598, Matorral del *Hammada hispanica*. 13/04/2014, M. Abdelaziz, M.J. Gómez, J. Lorite & F. Perfectti. Det.: J. Lorite.**;** Tize-N-Beckanm, 1968-07-06, MA247545-1, Gomez-Campo.

***Moricandia suffruticosa* (Desf.) Coss.** MOROCCO: Rif central, carretera Taza-Aknoul, 850 m., 34º23.836' N 3º54.413' W. Taludes margosos. 13/04/2014. Leg. M. Abdelaziz, M.J. Gómez, J. Lorite & F. Perfectti. Det.: J. Lorite. GDA62599**;** Tafraute: Anti Atlas mountains, 18 km from Tafraoute on road to Ait Baha, 11.5 km from Ameln, 29.73 º N / -8.85 º W, 1460 m, 2007-02-16, MA786486, S.L. Jury & T.M. Upson;
Anti Atlas mountains, road to Tata from Igherm, by villages of Tagadirt and Oued Anzrg, 29.94 º N / -8.52 º W, 1320 m, 2007-02-14, MA786225, S.L. Jury & T.M. Upson; Ouarzazate: Gorges du Dadès, 1750 m, 1985-01-06, MA302833, C. Blanché, J. Fernández Casas, J. Molero, J.M. Montserrat & A. Romo; Agadir: Souk-el-Tleta d´Afella Irhir, SALA23397, A. Charpin, J. Fdez. Casas, F. Jacquemoud & D. Jeanmonod;
TUNISIA: gobernación de Gabès: Metlaoui, gorges de Seldja, 34.33 º N / 8.32 º E, 250 m, 2009-03-25, MA796733, A. Herrero & al.; Matmata (Hadege), 1907-02-01, MA50238, C.J. Pitard; Gafsa, Leila, 1909-03-01, MA50239, C.J. Pitard.

***Rytidocarpus moricandioides* Coss.** MOROCCO: Rif central, carretera Taza-Aknoul, 652 m., 34º23.836' N 3º54.413' W. Taludes margosos. 13/04/2014. Leg. M. Abdelaziz, M.J. Gómez, J. Lorite & F. Perfectti. Det.: J. Lorite. GDA62600**;** Moulay Yacoub, 228 m., 34º7.452' N 5º12.248' W. Taludes margosos. 13/04/2014. Leg. M. Abdelaziz, M.J. Gómez, J. Lorite & F. Perfectti. Det.: J. Lorite. GDA62601

***Eruca pinnatifica* (Desf.) Pomel.** Marruecos, carretera Missour- Boulemane, 800 m., 33º2.144' N 4º4.083' W. Cultivos sobre suelos arenosos. 13/04/2014. Leg. M. Abdelaziz, M.J. Gómez, J. Lorite & F. Perfectti. Det.: J. Lorite. GDA62602

Figure S1

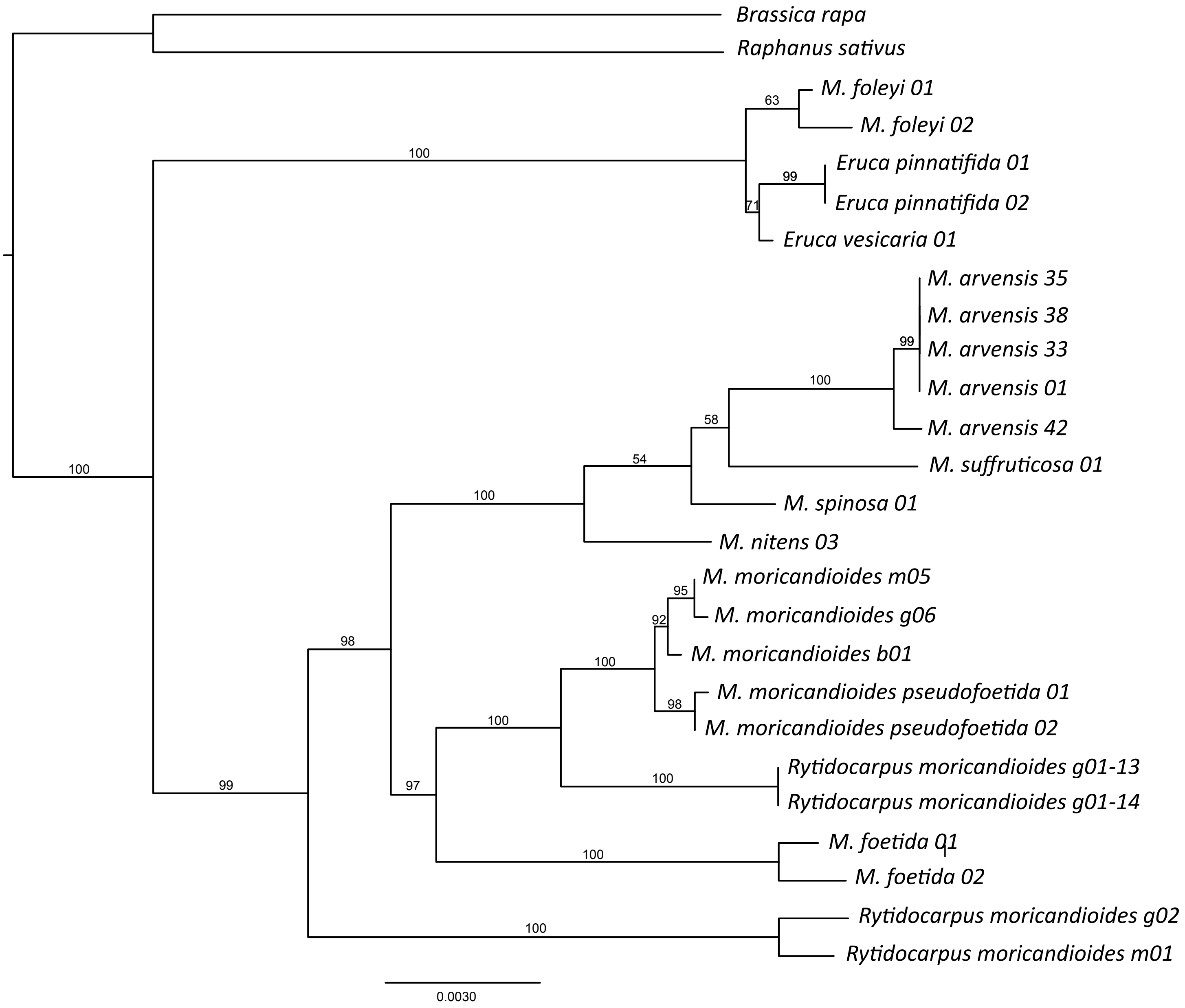
Brassica rapa
Raphanus sativus
M. foleyi 01
M. foleyi 02
Eruca pinnatifida 01
Eruca pinnatifida 02
Eruca vesicaria 01
M. arvensis 35
M. arvensis 38
M. arvensis 33
M. arvensis 01
M. arvensis 42
M. suffruticosa 01
M. spinosa 01
M. nitens 03
M. moricandioides m05
M. moricandioides g06
M. moricandioides b01
M. moricandioides pseudofoetida 01
M. moricandioides pseudofoetida 02
Rytidocarpus moricandioides g01-13
Rytidocarpus moricandioides g01-14
M. foetida 01
M. foetida 02
Rytidocarpus moricandioides g02
Rytidocarpus moricandioides m01
100
63
99
71
99
100
58
54
100
95
92
100
98
98
100
100
97
99
100
100
0.0030

Figure S2

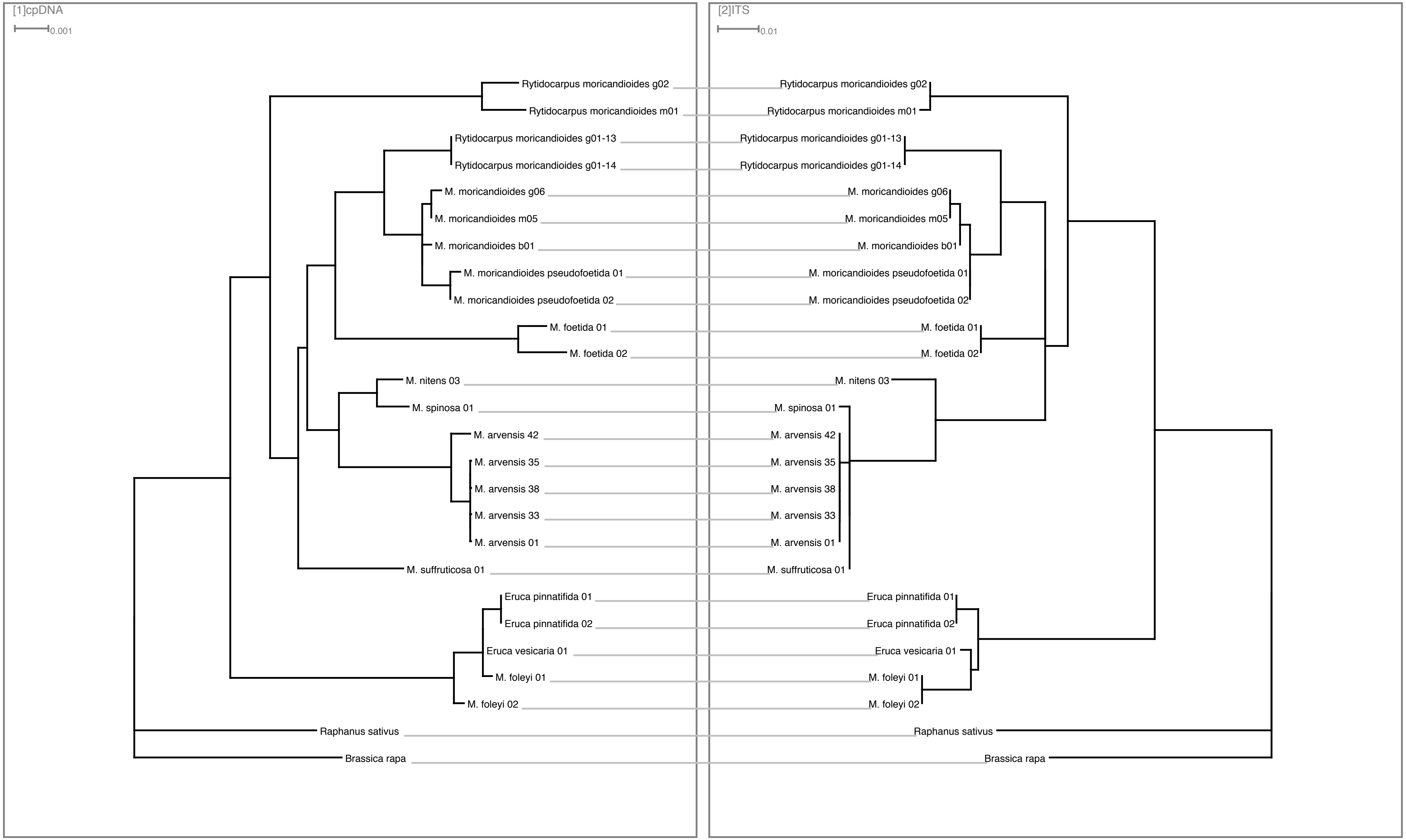
[1]cpDNA
0.001
[2]ITS
0.01
Rytidocarpus moricandioides g02
Rytidocarpus moricandioides m01
Rytidocarpus moricandioides g01-13
Rytidocarpus moricandioides g01-14
M. moricandioides g06
M. moricandioides m05
M. moricandioides b01
M. moricandioides pseudofoetida 01
M. moricandioides pseudofoetida 02
M. foetida 01
M. foetida 02
M. nitens 03
M. spinosa 01
M. arvensis 42
M. arvensis 35
M. arvensis 38
M. arvensis 33
M. arvensis 01
M. suffruticosa 01
Eruca pinnatifida 01
Eruca pinnatifida 02
Eruca vesicaria 01
M. foleyi 01
M. foleyi 02
Raphanus sativus
Brassica rapa

Figure S3

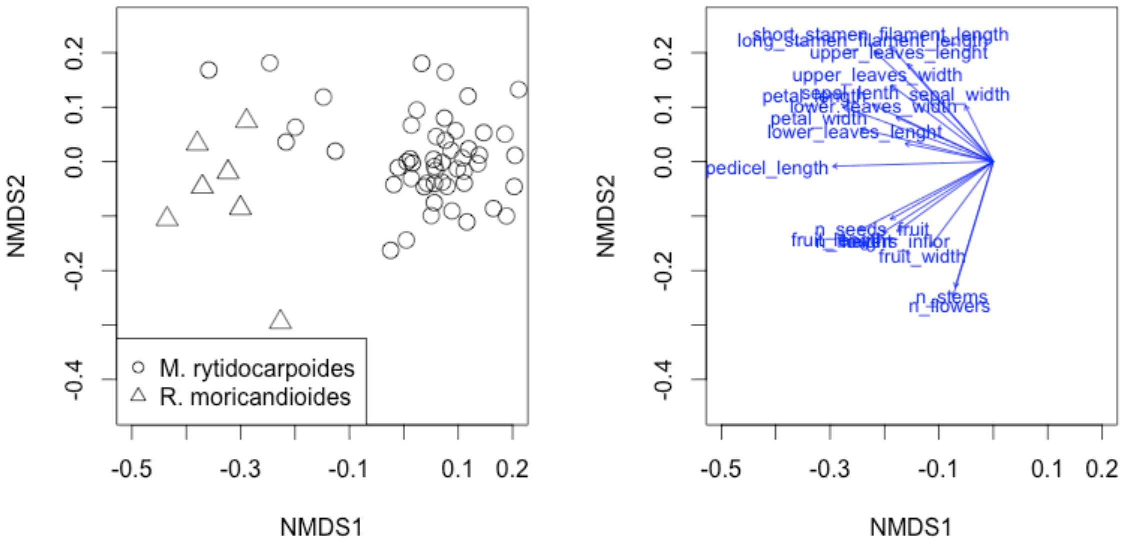
M. rytidocarpoides
R. moricandioides
NMDS1
NMDS2
-0.5
-0.3
-0.1
0.1
0.2
-0.4
-0.2
0.0
0.1
0.2
upper_leaves_lenght
upper_leaves_width
sepal_width
lower_leaves_width
petal_width
lower_leaves_lenght
pedicel_length
fruit_width
n_stems
n_flowers
NMDS1
NMDS2

Figure S4

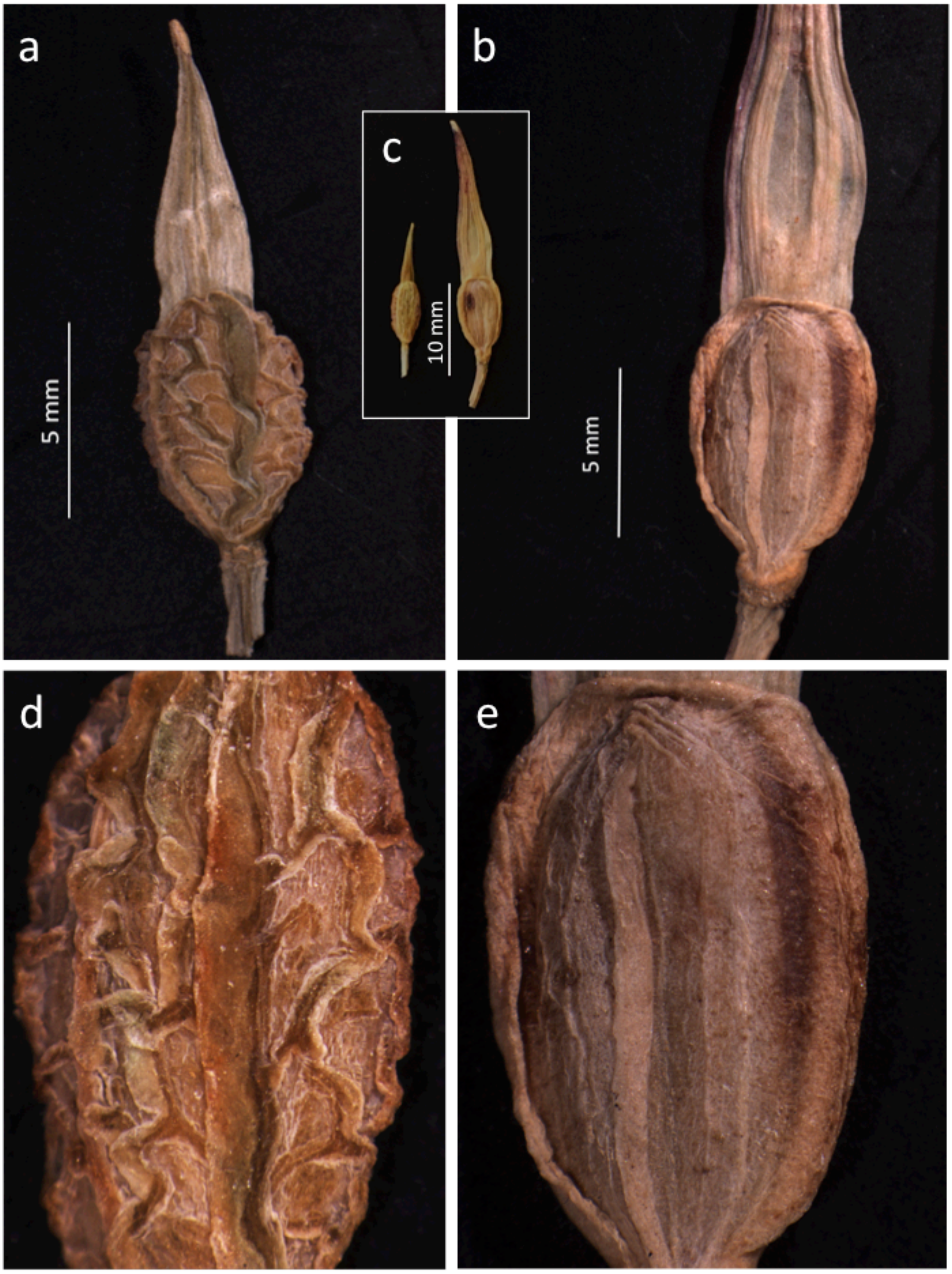

Supplement: Supplemental Information 1 — Supplementary information contains: Table S1: GenBank accessions. Table S2: Comparison between the proposed new species Moricandia rytidocarpoides and Rytidocarpus moricandioides (N. Africa) for several quantitative morphological traits. Table S3: Comparison of the proposed new species Moricandia rytidocarpoides with the most related species within the genus and with Rytidocarpus moricandioides from North Africa. Table S4: Additional examined specimens. Microsoft Word - Supplementary Material-Moricandia phylogeny-R01.docx Figure S1: Maximum likelihood tree produced with the complete 24-samples set. Branch labels represent bootstrap percentage values. Scale: mean expected rates of substitution per site. Figure S2: Tanglegram showing the maximum likelihood inference trees obtained from cpDNA (ndhF + trnT–trnF) and nuclear (ITS1 + ITS2) sequences. Figure S3: Biplots showing the result of non-metric multidimensional scaling (NMDS) for individuals (left) and variables (right) performed with the morphological data presented in Supplementary table 2. Model data in the left are referred to the result of a Permutational Multivariate analysis using species as factor. Figure S4: Microphotography of fruits of M. rytidocarpoides (left) and R. moricandioides (right). Fruits (A and B) and detail of fruit valve (C and D). Central insert (C) shows size differences of the fruit. [file peerj-05-3964-s001.pdf]
